# Gold nanostructure-enhanced immunosensing: ultra-sensitive detection of VEGF tumor marker for early disease diagnosis

**DOI:** 10.1038/s41598-024-60447-2

**Published:** 2024-05-07

**Authors:** Sadaf Yarjoo, Hossein Siampour, Mehrsa Khalilipour, Reza H. Sajedi, Hassan Bagheri, Ahmad Moshaii

**Affiliations:** 1 Department of Physics, Tarbiat Modares University, P.O Box 14115-175, Tehran, Iran; 2 Biosensor Research Center (BRC), Isfahan University of Medical Sciences, P. O. Box 81746-73461, Isfahan, Iran; 3 Department of Sensor and Biosensor, Faculty of Interdisciplinary Sciences and Technologies, Tarbiat Modares University, P. O. Box 14115-336, Tehran, Iran; 4 Department of Biochemistry, Faculty of Biological Sciences, Tarbiat Modares University, Jalal Ale Ahmad Highway, Tehran, 14115-154 Iran; 5 Chemical Injuries Research Center, Systems Biology and Poisonings Institute, Baqiyatallah University of Medical Sciences, Tehran, Iran

**Keywords:** Electrochemical immunosensor, VEGF detection, Porous gold electrode, Fabrication methodology, Biomarker measurement, Nanoscale devices, Biosensors

## Abstract

We present an advanced electrochemical immunosensor designed to detect the vascular endothelial growth factor (VEGF) precisely. The sensor is constructed on a modified porous gold electrode through a fabrication process involving the deposition of silver and gold on an FTO substrate. Employing thermal annealing and a de-alloying process, the silver is eliminated from the electrode, producing a reproducible porous gold substrate. Utilizing a well-defined protocol, we immobilize the heavy-chain (VHH) antibody against VEGF on the gold substrate, facilitating VEGF detection through various electrochemical methods. Remarkably, this immunosensor performs well, featuring an impressive detection limit of 0.05 pg/mL and an extensive linear range from 0.1 pg/mL to 0.1 µg/mL. This emphasizes it’s to measure biomarkers across a wide concentration spectrum precisely. The robust fabrication methodology in this research underscores its potential for widespread application, offering enhanced precision, reproducibility, and remarkable detection capabilities for the developed immunosensor.

## Introduction

Today, early diagnosis and prompt treatment of various cancers are very important, where delayed diagnosis directly leads to higher mortality rates and lower treatment costs^1,2^. According to WHO, tumors are responsible for 90% of human cancer cases and can emerge in various body regions, including the lungs, breasts, bladder, prostate, intestines, and kidneys. Vascular endothelial growth factor (VEGF) is recognized as a significant cancer biomarker, playing a crucial role in forming of cancer tumors^3^. Monitoring VEGF levels enables the estimation of tumor status in many cases.

Normally, the cancer threshold limit for the VEGF biomarker is 207 pg/mL in serum and 23 pg/mL in the blood plasma^3^. Of course, the VEGF threshold value may slightly change in various cancer types^3^. Increased levels of the VEGF biomarker have been observed in various tumor-associated cancers, including carcinoma, lung, colon, prostate, brain, kidney, and breast cancers^4–12^. Consequently, determining VEGF levels in clinical samples is a standard way for diagnosis, prognosis, and therapeutic monitoring of various cancers.

The detection of VEGF biomarkers can be achieved using different biosensor transducers like optical^13^, fluorescence^14^, field-effect transistor^15^, quartz crystal microbalance^16^, and surface plasmon resonance^17^. On the other hand, electrochemical immunosensors offer a highly sensitive and specific approach to detecting various antibody/antigen elements^18^. Electrochemical immunosensing of VEGF typically involves immobilizing VEGF-specific antibodies onto a transducer surface, which interacts with VEGF molecules in a sample, leading to a measurable electrochemical response^19^. This approach offers several advantages, such as rapid detection, minimal sample requirement, and the ability to detect VEGF in complex biological media^20^.

Generally, incorporating nanostructures into the electrode design of electrochemical biosensors has been shown to enhance the effective electroactive surface area, charge transfer, and thus the sensitivity of the electrodes^15,21^. Various nanomaterials, including gold^22^, silver^23^, graphene^24^, and nanocomposites^25^, have been employed in the design of the electrode surface. Among them, Au nanostructures are the preferred choice due to their high conductivity, biocompatibility, and resistance to rapid oxidation. Gold nanostructures can be deposited onto the surface using various methods such as physical vapor deposition (PVD), chemical vapor deposition (CVD), hydrothermal synthesis, and reducing agent synthesis^22–25^.

Dealloying and lithography are two distinct methods commonly employed in the creation of mesoporous gold nanoparticles. In the lithography method, gold is first incorporated into a molecular template, typically a copolymer such as PS-b-PEO (polystyrene–poly(ethylene oxide))^26^, which serves as a framework for structuring mesoporous gold nanoparticles. Lithography enables precise patterning and the creation of nanoparticles with various sizes, facilitating exact control over the size and shape of mesoporous gold nanoparticles. Generally, increased nanoparticle porosity enhances biosensor sensitivity^27^. However, lithography requires sophisticated equipment and technical expertise, making the process costly and time-consuming. In contrast, the dealloying method offers a relatively simple and efficient means for producing mesoporous gold nanoparticles. In our study, we have chosen to utilize the dealloying method for fabricating mesoporous gold nanoparticles, driven by its advantages, which include simplicity and acceptable efficiency. By utilizing the dealloying method, we aim to achieve satisfactory control over the structure and properties of mesoporous gold nanoparticles for biosensor template fabrication.

The current research, the main goal is to design and fabricate an Au nanostructured electrode for ultra-sensitive immounosensing detection of VEGF through various electrochemical methods. The nano-porous Au electrode has been fabricated using the simple and reliable dealloying method. Initially, thin films of silver and gold were deposited onto the fluorine-doped tin oxide (FTO) substrate using PVD, then the surface was modified through an annealing procedure. The dealloying method with HNO_3_ solution effectively removes the silver from the structure to obtain a nano-porous Au electrode.

By implementing a straight forward procedure for antibody immobilization on the gold substrate, VEGF detection was accomplished through cyclic voltammetry (CV) and electrochemical impedance spectroscopy (EIS). Finally, measurement of the VEGF antigen using the designed immunosensor has been carried out, and the sensor demonstrates a high linear detection range with a very low detection limit of 0.05 pg/mL.

## Experimental section

### Chemicals and materials

High-purity Au and Ag metallic pellets were used for the PVD process. These metals were deposited onto the FTO substrates (15 Ωsq − 1), which were cut into pieces of 0.8 × 1.25 cm^2^ with a thickness of 2 mm. In the experiments, phosphate-buffered saline (PBS) was used as a buffer solution with a pH of approximately 7.4. Additional chemicals used included mercaptoacetic acid (MAA), 1-Ethyl-3-(3-dimethylaminopropyl) carbodiimide (EDC), and N-Hydroxysuccinimide (NHS), all of which were obtained from Sigma–Aldrich. Also, gelatin, variable domain of heavy-chain antibody (VHH) against VEGF^28^, VEGF antigens^29^, and ethanol (99.9% purity) were utilized in the study.

For the electrochemical measurements, the following chemicals were used: potassium chloride (KCl), potassium ferricyanide (K_3_[Fe (CN)_6_]), potassium ferrocyanide (K_4_[Fe (CN)_6_]), and deionized water (DI water). These chemicals were utilized as received without further purification beyond their initial specifications.

### Instruments

The morphology of the fabricated electrodes was characterized by field emission scanning electron microscopy (FE-SEM) equipped with EDS (FEI Nova NanoSEM-450 model). X-ray diffraction (XRD) was used for phase crystalline identification (X’Pert Pro MPD system equipped with Cu-Kα radiation). A contact angle measurement device studied the wetting properties during the surface modification (Jikan, Iran) The CVand EIS were performed with the Origalysis potentiostat system (ElectroChem SAS, France) through a three electrodes electrochemical cell containing Ag/AgCl as a reference electrode, Au plate as a counter electrode, and the nano-porous gold electrode as the working electrode.

### Biosensors fabrications

**Figure 1:**
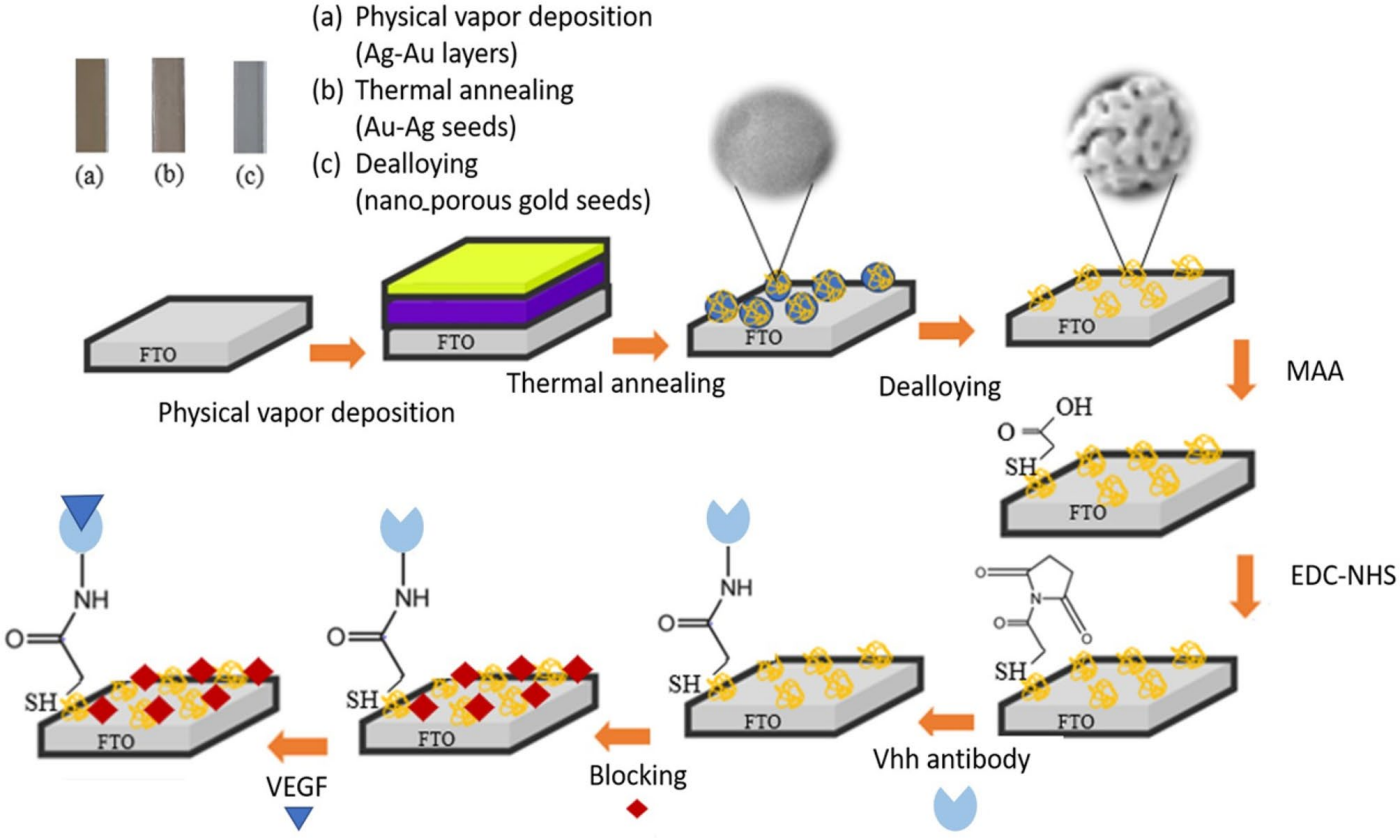
Schematic of the preparation of the Au dealloyed porous electrode, followed by various steps for immobilizing the antibody on the electrode, and finally, the immunosensing detection of VEGF.

We prepared the primary substrate before the physical vapor deposition by following a specific washing protocol. The surface of the FTO electrodes was scrubbed with acetone, ethanol, and deionized water. Subsequently, we ultrasonicated them for 15 min and then dried them inside an oven. After that, different thicknesses of the silver film were deposited on the FTO electrodes, followed by the deposition of a 5 nm gold thin film, all by the PVD method. The pressure in the PVD chamber was maintained at 1 × 10^–6^ Torr before the deposition started. Also, the growth rate for deposition was adjusted to 0.1 nm/s, and thin film thickness was controlled by quartz crystal microbalance. To fabricate an Ag-Au alloy nanostructure, we subjected the electrodes with an Ag-Au thin film to a thermal annealing process in a furnace for 2 h at the temperature of 550 °C. Afterward, to modify the electrode, the annealed electrode was dipped into 65% nitric acid at room temperature for 15 min to induce dealloying and remove the Ag material from the electrode. Finally, the modified electrode was thoroughly rinsed with DI water. We followed a series of steps to biofunctionalize the gold surface with antibody. First 50 µL of an MAA solution (14 mmol/L) was applied to the working electrode at room temperature for 2 h. Then, the electrode was washed with ethanol to remove any unbound MAA from the surface. To activate the carboxylic groups, a solution of 50 μL of 50 mM EDC/NHS (1:1) in PBS buffer at pH 6.0 was placed on the working electrode surface for one hour at room temperature. Next, 50 μL of 10 µg/mL VHH anti-VEGF was deposited on the activated electrode overnighted at 4 ℃ for antibody fixation. To prevent and block non-specific bindings, we applied a gelatin solution (50 μL of 20 mg/mL in PBS) to the electrode for 45 min^30^. Subsequently, different VEGF concentrations were introduced to the activated surface for 45 min at 4 ℃ to characterize the biosensor. The VEGF solution is prepared in PBS at various concentrations. Throughout all procedures, the electrode was kept in a dark ambient environment to prevent unwanted photochemical degradation of the thiol component of MAA^31–33^. After activating the carboxylic groups, any unbound reagents were washed away with a PBS solution (pH 7.4) at each stage. For a visual representation of the fabrication of the sensor, the antibody fixation and antigen detection stages are all shown in Fig. 1.

## Result and discussion

### Characterization of the electrode surface

**Figure 2:**
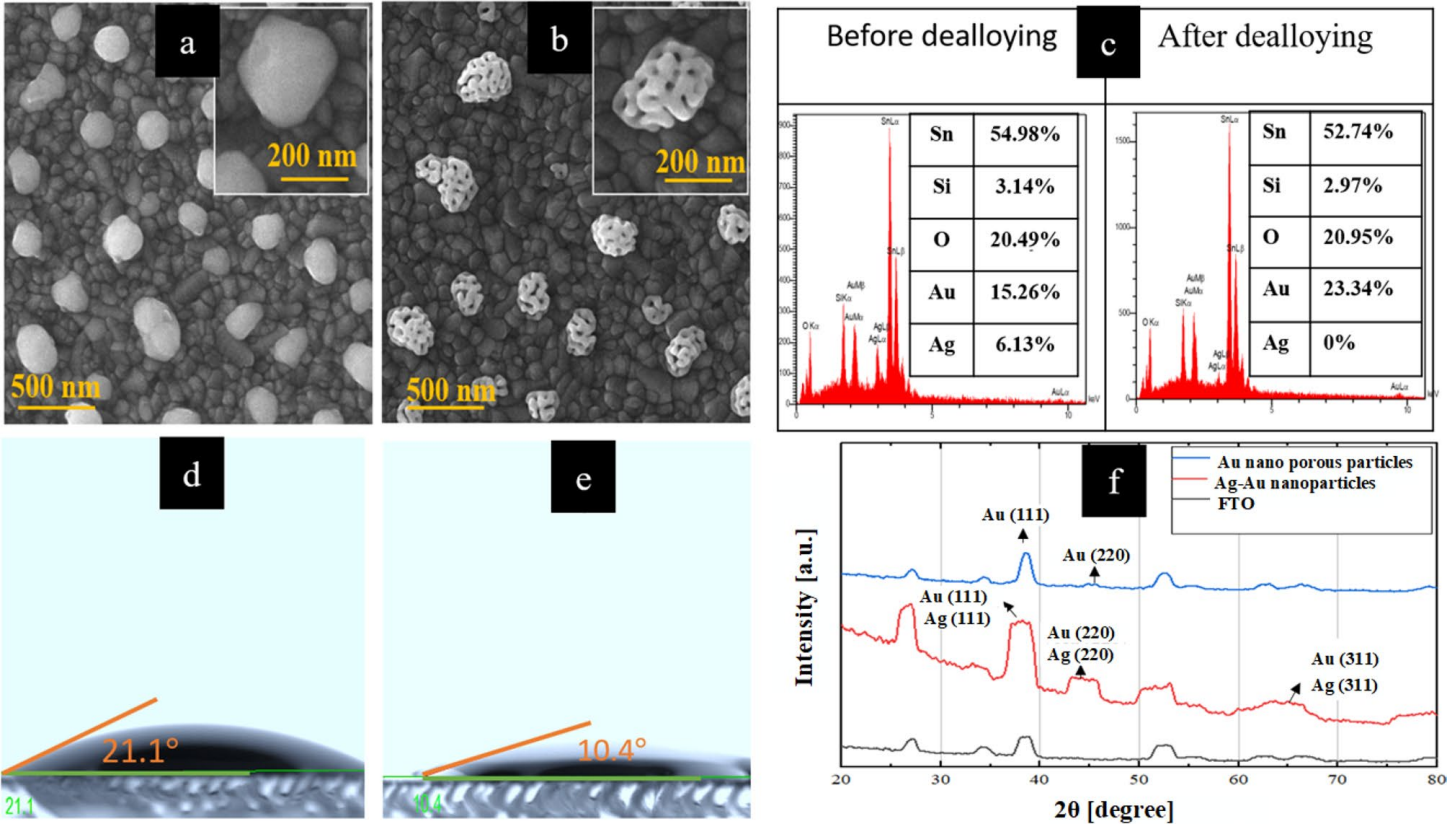
(**a**) Morphology of the Ag-Au nanoparticles after the annealing process, (**b**) Morphology of the Au nano-porous structures after the dealloying process, (**c**) EDS quantitative result of the Ag-Au nanoparticles before and after dealloying. Especially, after dealloying, all silver was removed. (**d**) The contact angle measurement for the optimized electrode after the annealing process, with measured contact angle of 21.1°, and after the dealloying process (**e**) with the measured 10.4°. The graph (**f**) shows XRD pattern of the FTO, the Ag-Au alloy and the Au dealloyed electrode.

Figure 2 illustrates the different electrodes fabricated through alloying and dealloying processes. Figure 2a shows the morphologies of the alloying structure of Ag-Au on the FTO substrate. Due to the thermal annealing process, semi-spherical Ag-Au nanoparticles are shaped on the FTO surface, wherein a hybrid layer containing a 25 nm silver thin film and a 5 nm gold thin film were initially deposited using the PVD system. In Fig. 2b, the Au nano-porous structures are visible after dealloying. The energy-dispersive X-ray spectroscopy (EDS) confirms the complete removal of silver during the dealloying process, as shown in Fig. 2c.

Figures 2d and e show the difference in hydrophilicity of the optimized Ag-Au electrode before and after the dealloying process, as demonstrated via the contact angle measurement. The porosity of the formed gold nanostructures directly affects the measured contact angle, as the presence of nanopores on the surface increases the hydrophilicity of the working electrode. After dealloying, the contact angle decreased from 21.1$^\circ$ to 10.4$^\circ$, indicating that the surface had become more hydrophilic. This almost 50% decrease in the contact angle demonstrates improved hydrophilicity of the formed structure. Consequently, the low contact angle of the electrode surface leads to better connecting biomolecules to the electrode, enhancing the biofunctionality and detection capability of the sensor. To confirm the phases and components of the optimized electrode, XRD patterns were conducted, and the results are shown in Fig. 2f. In addition, the XRD patterns of the bare FTO electrode, the bi-layer of 25 nm Ag and 5 nm Au electrode annealed at 550 °C, and the final nano-porous gold nanostructures have been shown for comparison purposes.

The XRD patterns provide evidence for creating Ag-Au nanoparticles and the porous gold particles on the FTO substrate. The peaks of Au, Ag, and FTO have many similar diffraction angles, leading to several broadened peaks appearing in the XRD spectrum. The crystallographic directions of various planes have also been displayed in the spectrums. The primary crystallographic orientations for the FTO substrate correspond to the (110), (101), (200), (211), (310), and (301) planes, with corresponding 2$\uptheta$ values of 26.6$^\circ$, 33.8$^\circ$, 37.8$^\circ$, 52$^\circ$ and 62$^\circ$, respectively. We observe even stronger peaks for the gold and silver nanoparticles. The prominent peaks of Ag-Au nanoparticles are observed at 2$\uptheta$ values of 38.2$^\circ$, 64.6$^\circ$, and 77.6$^\circ$ with the miller indices (hkl) of (111), (220), and (311) for the primary crystallographic orientations. After dealloying, nano-porous gold nanoparticles remain with the plane orientations of (111) and (220) at 2$\uptheta$ the angles of 37.5$^\circ$ and 43.6$^\circ$, respectively.

Two important parameters determine the size and porosity of porous nanostructures during the alloying and dealloying processes: the ratios of gold to silver in the PVD process and the annealing temperature in the thermal processing.

**Figure 3:**
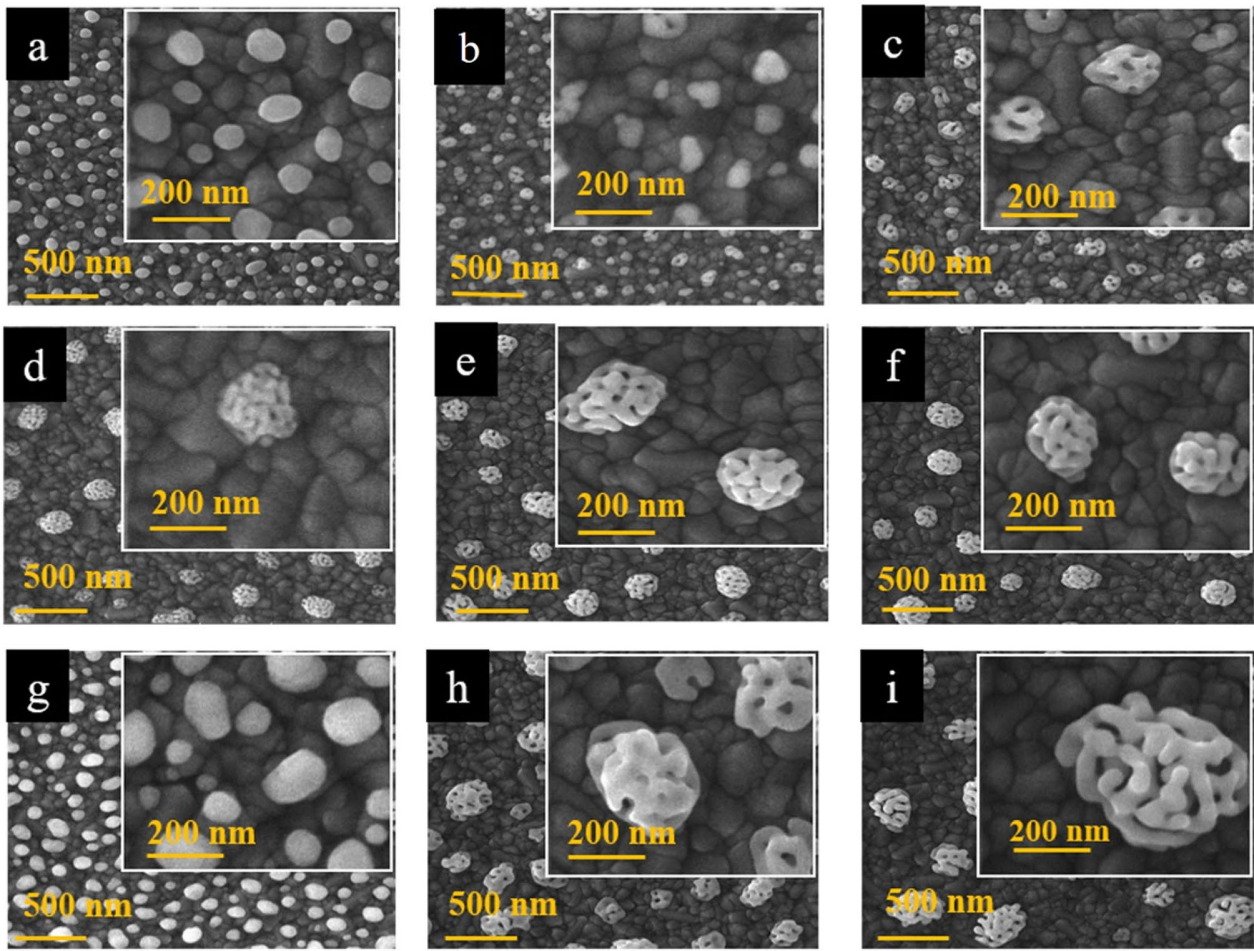
FE-SEM images of the nano-porous gold with different gold and silver thicknesses with the same annealing temperature of 550 °C, including: (**a**) 5 nm Ag@ 5 nm Au, (**b**) 10 nm Ag@ 5 nm Au, (**c**) 15 nm Ag@ 5 nm Au, (**d**) 20 nm Ag@ 5 nm Au, (**e**) 25 nm Ag@ 5 nm Au, (**f**) 30 nm Ag@ 5 nm Au, (**g**) 5 nm Ag@ 10 nm Au, (**h**) 20 nm Ag@ 10 nm Au, (**i**) 40 nm Ag@ 10 nm Au.

Figure 3 shows the FE-SEM images of fabricated gold nanostructures with different thickness ratios of Ag and Au layers after the dealloying process at the same annealing temperature of 550 °C. The figure shows that the thickness ratio of silver to gold considerably affects the size of the dealloyed nanostructures. No observable porosity was revealed after post-dealloying in the case of 5 nm silver and 5 nm gold in Fig. 3a. Conversely, as the silver thicknesses increase to 10, 15, 20, 25, and 30 nm, a corresponding boost in the porosity of the emerged post-dealloying structure, is revealed. However, there is a small difference between the FE-SEM results of Fig. 3e and f, corresponding to the silver thicknesses of 25 nm and 30 nm, respectively. The supplementary CV and EIS characterizations of these substrates do not show a significant difference (as demonstrated in Fig. S1). This conducts us to select the case of 25 nm silver and 5 nm gold arrangement as the best initial Ag-Au thicknesses for the alloy-dealloy process.

In continuation of Fig. 3 results, the morphological structures of various arrangements involving 10 nm gold thin film integrated with different silver thicknesses of 5, 20, and 40 nm were investigated, as shown in Fig. 3g–i. Again, no porosity was exhibited after dealloying in the case of the 10 nm gold thin film coupled with the 5 nm silver thin film. As observed previously, raised silver content correlates with increased porosity. Drawing from this analysis of these figures, one can infer that the thickness of the initial silver substrate compared to the final gold overlayer plays a crucial role in initiating and boosting the porosity of the structure. Simultaneously, the amount of gold dictates the ultimate dimensions of the formed nanostructure. However, the use of more than 10 nm gold thickness was generally limited due to the increased gold consumption, which posed economic inefficiency concerns.

To investigate and obtain an optimized annealing temperature for the thermal process, the morphologies and the average sizes of nanostructures after the dealloying process were measured as a function of the annealing temperature. As the temperature increases, the porosities become more apparent, and their surface distribution increases in the final structure. Despite this issue, according to the size distribution histograms, the overall size structure is similar for temperatures of 550 and 600 °C. Therefore, the electrode with an annealing temperature of 550 °C with and 25 nm silver and 5 nm gold thicknesses were selected as the optimized electrode for further sensing investigations. In addition, further investigation results have been shown in Fig. S2 in the supplementary information.

### Electrochemical characterization of the fabricated electrodes

**Figure 4:**
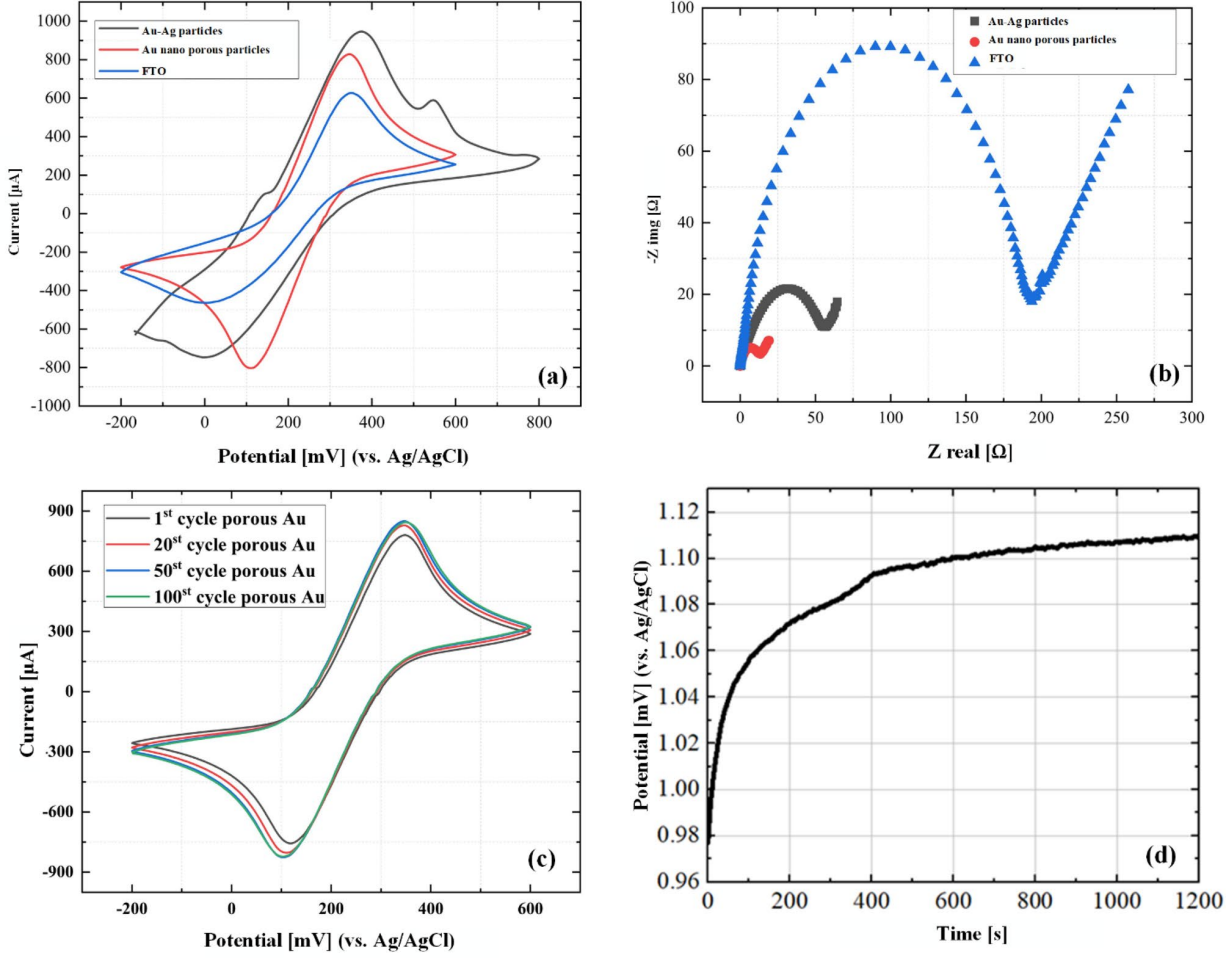
Electrochemical diagrams of various modification step including (**a**) CV, (**b**) EIS, (**c**) Investigating the stability of the modified electrode after the first, 20th, 50th, and 100th cycle of CV, and (**d**) Open circuit potential of the Ag-Au nanoparticles over the first 1200 s of exposure to the HNO_3_ solution.

In this section, the modified electrodes were subjected to electrochemical characterization via CV and EIS. The results for the bare FTO, the optimized Au–Ag electrode, and the optimized Au–Ag electrode after the dealloying process were compared in Fig. 4a and b. Based on Fig. 4a, it was determined that the peak-to-peak interval potentials of the dealloyed electrode and the bare FTO electrode are 235 mV and 350 mV, respectively. This means that the peak-to-peak potential of the dealloyed electrode is approximately 33% less than that of the bare FTO. Additionally, the maximum current of 624 µA was achieved for the bare FTO electrode, while this peak current increased to 820 µA for the dealloyed electrode.

As shown in Fig. 4a, the CV curve shows three substantial peaks in the forward scan. The first cathodic peak at 0.141 V relates to surface-confined processes of the Ag surface, and the second cathodic peak at 0.385 V relates to oxidation of the [Fe(CN)_6_]^–3/–4^ redox system at the Au surface. The peak at 0.552 V corresponds to the oxidation of the Ag surface on the FTO with a significant current. In the backward scan, the first cathodic peak relates to reducing the [Fe(CN)_6_]^–3/–4^ redox system at the Au surface at 0.05 V.

The difference between the CV curves of the porous Au electrode and the Ag-Au electrode in Fig. 4a can be explained in terms of the effect of Ag nanoparticles on the electrochemical current response. The electrochemical current of the Ag-Au electrode involves the redox currents of both silver and gold as they interact with the electrolyte solution^34,35^. Due to the process of dealloying, the silver structure is removed, resulting in a decrease in the redox current response, possibly corresponding to a lack of silver contribution in the electrochemical reactions.

According to the EIS result, the FTO surface resistance was about 194 Ω. In this case, the surface resistance reaches 56 Ω, indicating about a 72% decrement relative to that of the bare FTO electrode.

In the next step, silver was dealloyed and the porous Au nanoparticles were produced.

This indicates better reversibility of the dealloyed electrode against redox reactions relative to the same case before dealloying. Finally, the resistance of the modified dealloyed surface relative to the FTO surface decreased by 93%, reaching about 14 Ω. In addition, the stability of the electrode was investigated in the first, 20th, 50th, and 100th cycles, and it is shown in Fig. 4c that no significant change was seen after the 50th cycle. In order to gain insight into the time of the dealloying process, we measured the open circuit potential (OCP) to evaluate the impact of nitric acid dealloying on the electrode surface. As illustrated in Fig. 4d, after approximately 10 min, nearly 94% of the change of OCP has taken place. When the OCP stabilizes, dealloying process is fully developed, and all the silver is completely removed. In our experiments, we have chosen an alloying time of approximately 15 min to ensure the complete removal of silver from the electrode surface.

In Fig. S3, the influence of the annealing temperature on the electrochemical response of the modified electrode at three annealing temperatures of 450, 550, and 600 ℃ has been investigated. The diameter of the semicircle in the Nyquist plot represents the charge transfer resistance (${{\text{R}}}_{{\text{ct}}}$). The ${{\text{R}}}_{{\text{ct}}}$ at the temperatures of 450 and 600 $^\circ{\rm C}$ is higher than that of 550 $^\circ{\rm C}$. The same scenario is also repeated for the peak current of the 550 $^\circ{\rm C}$ sample, in the CV results.

As depicted in Fig. S4, it is apparent that the square root of the scan rate shows a clear linear relationship with the anodic peak current. This observation strongly indicates that the redox reaction involving the nano-porous gold nanostructures follows a diffusion-controlled process. The Randles–Sevcik equation is commonly used to determine the effective surface area of the electrode:

1 $$ I_{p} = 2.69 \times 10^{5} n^{3/2} AC_{0} D^{1/2} v^{1/2} , $$

where, Ip is the peak current (A), n is the number of electrons transferred in the reaction, A is the effective surface area (cm^2^), D is the diffusion coefficient (cm^2^/s), C_0_ is the initial bulk concentration of the electroactive species (mol/cm^3^), and *ν* is the scan rate (V/s)^36,37^. To evaluate the effective electrode surface area of the annealed Au electrode and that of the dealloyed electrode, the CV curves are measured in KCl solution (0.1 M) containing the redox couple [Fe(CN)_6_]^–3/–4^ (2.5 mM) for different scan rates. Figure S5 represents the results for I_p_ versus ν^1/2^. The effective surface area of the annealed Au electrode and the dealloyed electrode are obtained accordingly as 1.42 cm^2^ and 1.72 cm^2^, respectively. These results indicate a significant boost in the accessible surface area of the electrode through the dealloying process. More detailed results are also summarized in Table S1.

### Immunosensing characterization and analytical performance

**Figure 5:**
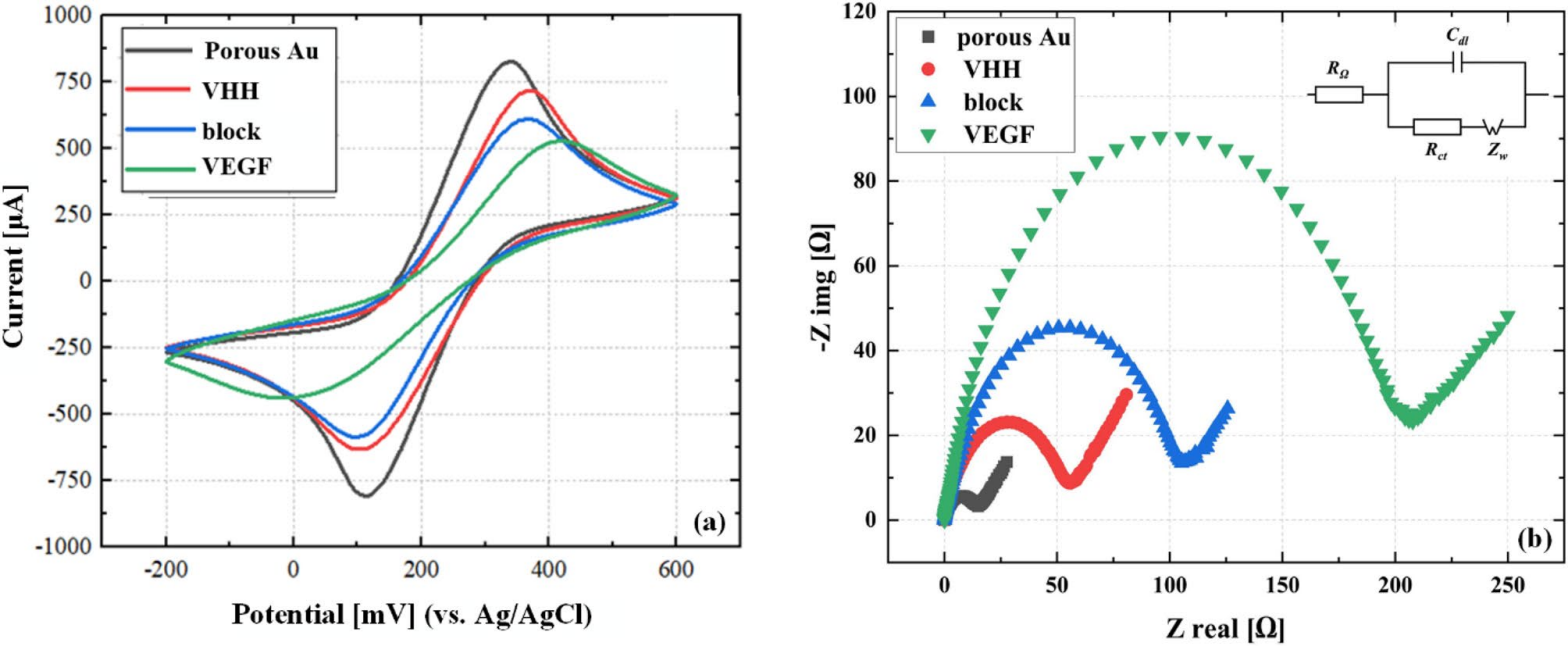
Various electrochemical plots including (**a**) CVs with a scan rate of 50 mV/s, (**b**) Nyquist curves, all were employed in a solution containing 2.5 mM [Fe (CN)_6_]^–3/–4^ to monitor the stepwise immobilization process of biological agents on the Au nano-porous electrode. The EIS plots from left to right corresponded to the stages of the Au nano-porous electrode, immobilization of VHH, gelatin blocking, and VEGF molecule capture on the electrode. Additionally, consistent color coding was used across all figures for clarity and consistency. The concentration of VEGF in this study was 1 pg/mL.

As previously mentioned in Fig. 1, the process of anchoring antibodies to the modified electrode and detecting the VEGF biomarker involves using MAA, EDC, and NHS. These compounds are utilized for immobilization on the modified electrode, facilitating the covalent attachment of antibodies to the transducer element. As shown in Fig. 5, the immunosensensing characterization was performed using CV and EIS measurements. First, the CV and EIS tests of the bare dealloyed electrode were performed. We conducted these tests after immobilizing the antibody onto the surface. The predominant covalent bonding of the VHH to the electrode surface leads to a further reduction in the anodic peak current to 717 $\mathrm{\mu A}$. After that, to block the empty places on the surface, we used gelatin, appearing in the increment of the electrode resistance from (56 Ω) for the antibody state to (107 Ω) after the block stage. In this case, the reduction of the current peak reads from (717 µA) to (610 µA). In the last step, VEGF antigen was placed on the surface to ensure the correct connection of all steps. As it is clear in the figure, in the last stage, which is also the antigen detection stage, the current peak in the CV diagram has decreased to (525 µA) in addition to about 31% increment of the peak-to-peak voltage compared to the state of Au porous. The EIS graph shows the resistance increment from (107 Ω) to (204 Ω), which are all proofs of antigen detection by the desired immunosensor.

Also, the result of the numerical values of the electrical parameters corresponding to an equivalent EIS circuit was used, and the results of the obtained parameters have been summarized in Table S2. Additionally, the equivalent circuit for modeling the EIS result has been shown in the inset of Fig. 5b.

**Figure 6:**
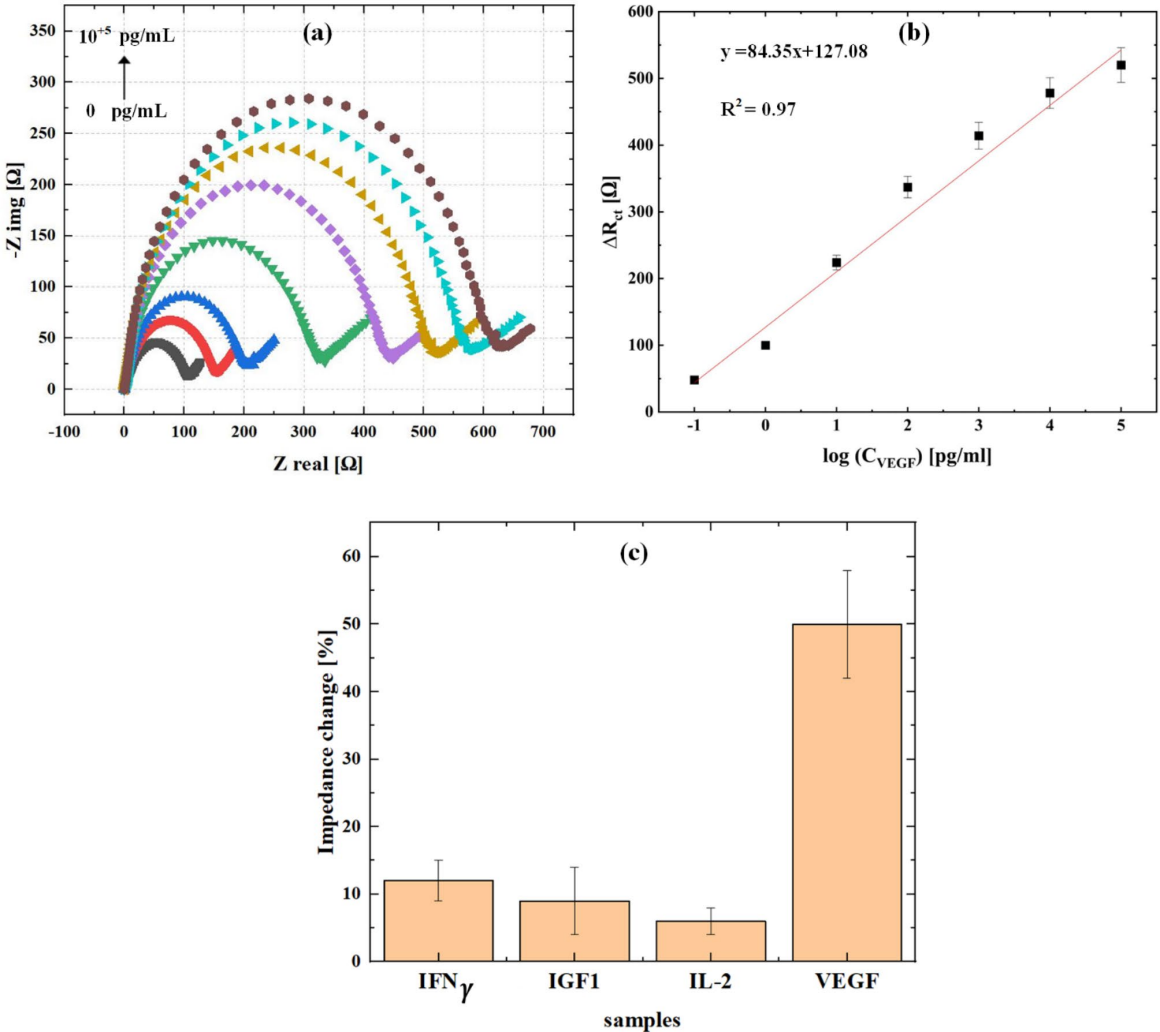
(**a**) The EIS responses of various VEGF concentrations ranging from 10^–1^ to 10^5^ pg/mL, (**b**) calibration curve response measured in a 2.5 mM [Fe (CN)_6_]^–3/–4^ solution and (**c**) selectivity test by using several interfering samples.

Figure 6 shows the calibration curve result of the VEGF determination by the fabricated sensor. In each step, the difference between the resistances ($\Delta {{\text{R}}}_{{\text{ct}}}$) and the state where VEGF is not present in the solution is measured to find the linear range. The equation of concentrations ranges from 0.1 pg/mL to 0.1 µg/mL can be expressed as ∆R_ct_(Ω) = 84.35Log (C_VEGF_) [pg/mL] + 127.08 with a regression coefficient of 0.97. This immunosensor has a very good limit of detection of 0.05 pg/mL. The Limit of Detection (LoD) value for the dealloyed Au electrode was calculated using the formula LoD = 3.3 σ/S. In this formula, σ represents the standard deviation of the blank response (for three measurements), and S is the slope of the calibration plot. Based on the slope of the calibration plot in Fig. 6b, the Limit of Detection (LoD) value was determined to be 0.05 pg/mL. It should be noted that due to the use of a semilogarithmic plot, the LoD equation should be redefined as follows^38–40^:

2 $$LOD=3.3\frac{\upsigma }{S},$$

3 $$\sigma =\sqrt{\frac{\sum {\left({y}_{i}-\overline{y }\right)}^{Y}}{deegree\,\, of \,\,freedom},}$$

4 $$S=2.303\frac{1}{x}\frac{dy}{d{log}^{x} },$$

5 $$LoD=3.3\frac{2.303 \sigma x}{\frac{dy}{dlo{g}_{10}^{x}}}.$$

In Fig. 6b, x is measured at 0.1 pg/mL, with a slope of 84.35 in log scale ($\frac{dy}{dlo{g}_{10}^{x}}=84.35)$ and $\sigma =6.23$. The limit of detection (LoD) is calculated to be 0.05 pg/mL.

To evaluate the reproducibility of the immunosensor, an experiment was conducted where the peak current response was examined across five electrodes fabricated under identical conditions. The objective was to determine whether the immunosensor could consistently deliver similar reliable results. The outcomes were presented in Fig. S6a, showing the relative standard deviation (RSD) of the current response to be below 5%, indicating high reproducibility of the immunosensor. Additionally, the cyclic voltammetry response of the immunosensor, as depicted in Fig. S6b of the Supporting Information, reveals minimal variation in peak current even over four weeks. This suggests the high stability of the immunosensor over an extended duration, highlighting its long-term durability.

Also, in Fig. 6c, the selectivity of our immunosensor is demonstrated. It shows that the interference species produce currents that are lower than 20% of the VEGF signal. The concentration of VEGF is equal to the concentrations of interfering species such as IFNγ, IGF1, and IL-2, all of which are at 1 pg/mL. This finding indicates the high-level selectivity of this sensor for detecting VEGF, as it suggests minimal interference from other substances.

To ensure the accuracy and precision of the immunosensor in human samples, we conducted a series of recovery experiments by applying the immunosensing test to three human samples, each spiked with known VEGF concentrations of 1 pg/mL, 100 pg/mL, and 1 ng/mL. The recovery tests were conducted based on the EIS scheme, and the findings indicated that the complex biological compounds in blood serum did not interfere with our results. We achieved successful recoveries with acceptable relative errors. Recoveries of 98%, 95.6%, and 96% were attained for the VEGF concentrations added at 1 pg/mL, 100 pg/mL, and 1 ng/mL, respectively. This demonstrates the reliability and accuracy of the immunosensor, further supported by the data of presented immunosensor in Table S3.

In Table 1, the characteristics of different biosensors for VEGF in the literature have been compared by this sensor. The findings illustrate that the nano-porous Au electrode outperforms various biosensors, especially regarding the limit of detection (LoD) and linear response range for VEGF detection.

**Table 1:** Comparison of the analytical capabilities of various VEGF immunosensors documented in the literature with the current research.

Biosensor substrate	Detection method	Linear range	LoD	Refs
Au/3-MPA/EDC-NHS/VEGF-R1	CV EIS	10–70 pg/mL	38 pg/mL	^2^
POLY (3, 4-ethylene dioxythiophene) (PEDOT)/Gold Nanoparticle (Au NP) Composites	EIS	1–20 pg/mL	0.5 pg/mL	^41^
PdPtMo CME NPs	EIS	10–10^6^ pg/mL	8.2 pg/mL	^42^
RGO/Au NPs/11MUA/Antibody/VEGF/HRP (Sandwich type)	CV SWV EIS	2–20,000 ng/mL	6 fg/mL	^43^
Cellulose paper	Fluorescence control (FApt)	100–5000 ng/mL	137 ng/mL	^44^
SPE/PANI/CNT/APT	DPV	0.5–10 $\times {10}^{6}$ pg/mL	0.4 pg/mL	^45^
GCE/GNRs-AuNPs/VEGF/ Ab-AuNPs@PoPD	CV EIS	0.500–500 ng/mL	300 pg/mL	^46^
Au nano-porous/MAA/EDC-NHS/VHH/Gelatin/VEGF	CV EIS	${10}^{-4}{-10}^{2}$ ng/mL	0.05 pg/mL	This work

## Conclusion

In this study, we have successfully designed an electrochemical immunosensor for detecting the VEGF tumor marker based on a modified porous gold electrode. The electrode is created through a series of straightforward processes involving silver and gold deposition on an FTO substrate, followed by thermal annealing and dealloying to eliminate silver from the electrode surface entirely. Employing a comprehensive protocol for antibody attachment to the gold substrate, we conducted VEGF detection using CV and EIS. Impressively, this immunosensor exhibits a remarkable detection limit of 0.05 pg/mL, making it an ideal tool for highly sensitive VEGF tests. Furthermore, the linear range extends from 0.1 pg/mL to 0.1 µg/mL, indicating not only a low limit of detection but also a wide dynamic range for accurate measurements.

In conclusion, this research not only establishes a robust method for sensitive electrode fabrication but also highlights its suitability for widespread adoption, positioning it as a promising candidate for scaling up in various scientific and medical contexts. The presented immunosensor offers several notable advantages for VEGF biomarker detection. However, it is essential to consider its potential limitations for practical applications, which include a relatively complex and costly fabrication process, antibody instability for long-term use, and a sophisticated production line for mass production. These factors may raise concerns regarding scaling up its fabrication.

## Supplementary Information

Supplementary Information.

## Data Availability

The datasets used and/or analysed during the current study available from the corresponding author on reasonable request.
