# Supplementary material for: Gold nanostructure-enhanced immunosensing: ultra-sensitive detection of VEGF tumor marker for early disease diagnosis
Source: Sci Rep. 2024 May 7;14:10450. doi: 10.1038/s41598-024-60447-2 (PMC11608251; doi:10.1038/s41598-024-60447-2)
Supplement: Supplementary file 1 — Supplementary Information. [file 41598_2024_60447_MOESM1_ESM.docx]

**Gold Nanostructure-Enhanced Immunosensing: Ultra-Sensitive Detection of VEGF Tumor Marker for Early Disease Diagnosis**

Sadaf Yarjoo^1^, Hossein Siampour^1,2^, Mehrsa Khalilipour^1^, Reza .H Sajedi^4^, Hassan Bagheri^5^, Ahmad Moshaii^1,3^*

^1^ Department of Physics, Tarbiat Modares University, Tehran, P.O Box 14115-175, Iran

^2^ Biosensor Research Center (BRC), Isfahan University of Medical Sciences, P.O.Box: 81746-73461, Isfahan, Iran

^3^ Department of Sensor and Biosensor, Faculty of Interdisciplinary Sciences and Technologies, Tarbiat Modares University, P.O.Box: 14115-336, Tehran, Iran

4 Department of Biochemistry, Faculty of Biological Sciences, Tarbiat Modares University, Jalal Ale Ahmad Highway, Tehran 14115-154, Iran

^5^Chemical Injuries Research Center, Systems Biology and Poisonings Institute, Baqiyatallah University of Medical Sciences, Tehran, Iran

*Email: [moshaii@modares.ac.ir](mailto:moshaii@modares.ac.ir)


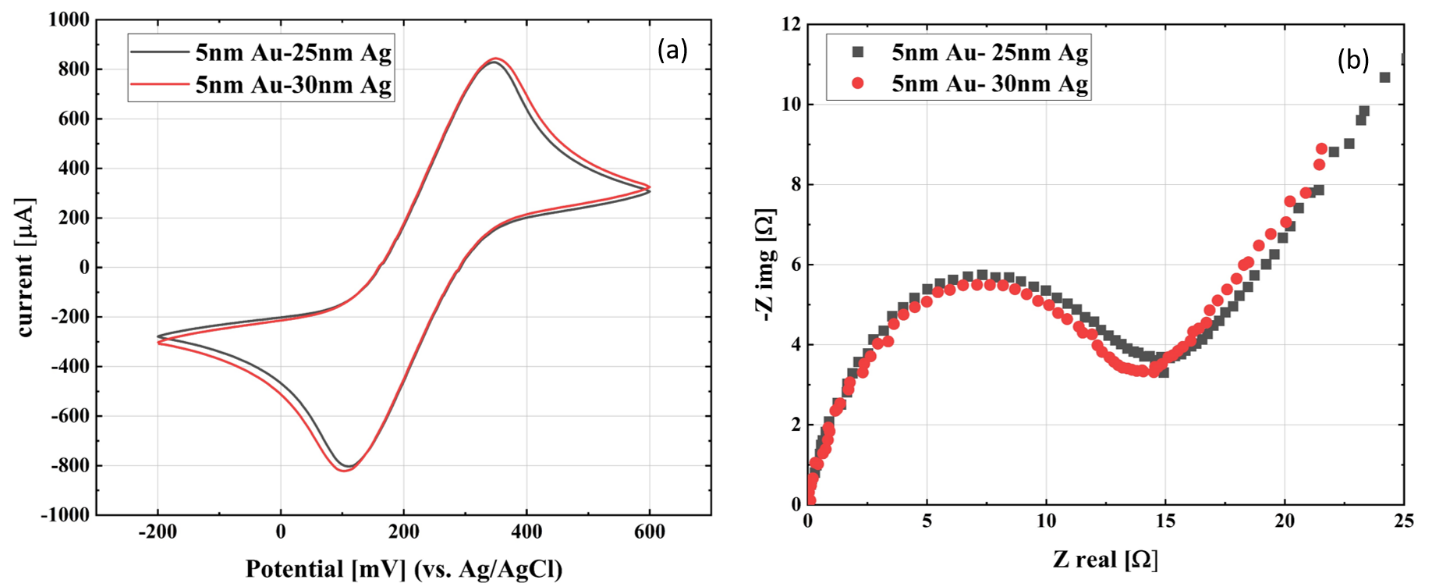


Figure S1: The CV (a), and EIS (b) characterization results of the nano-porous gold with different gold and silver thicknesses of 25 nm Ag@ 5 nm Au, and 30 nm Ag@ 5 nm.


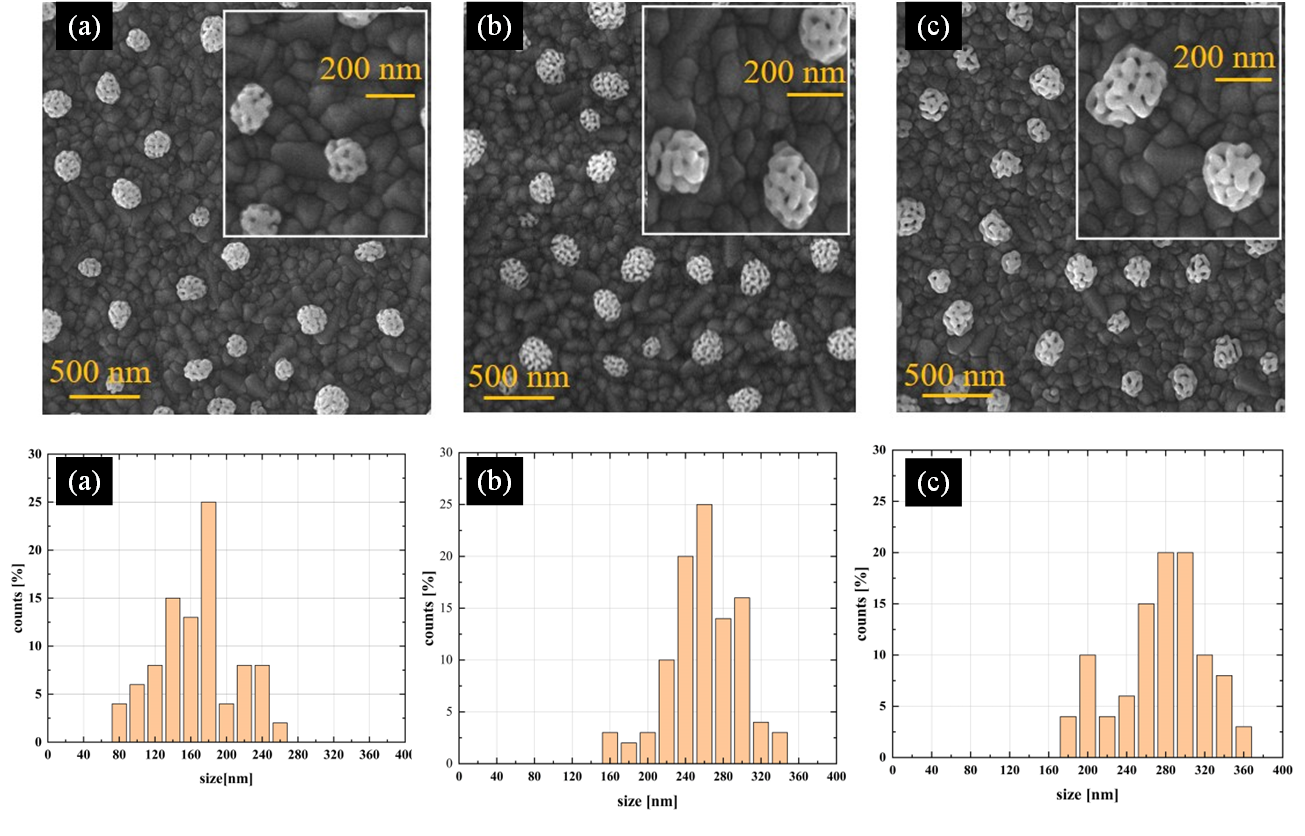


Figure.S2: FESEM images and histogram of different sizes of the formed nanostructures after the thermal annealing for the temperatures of a) 450$℃$, b) 550$℃$, c) 600$℃$. The thicknesses of gold and silver layers in all cases are fixed at 5nm gold and 25nm silver, respectively.


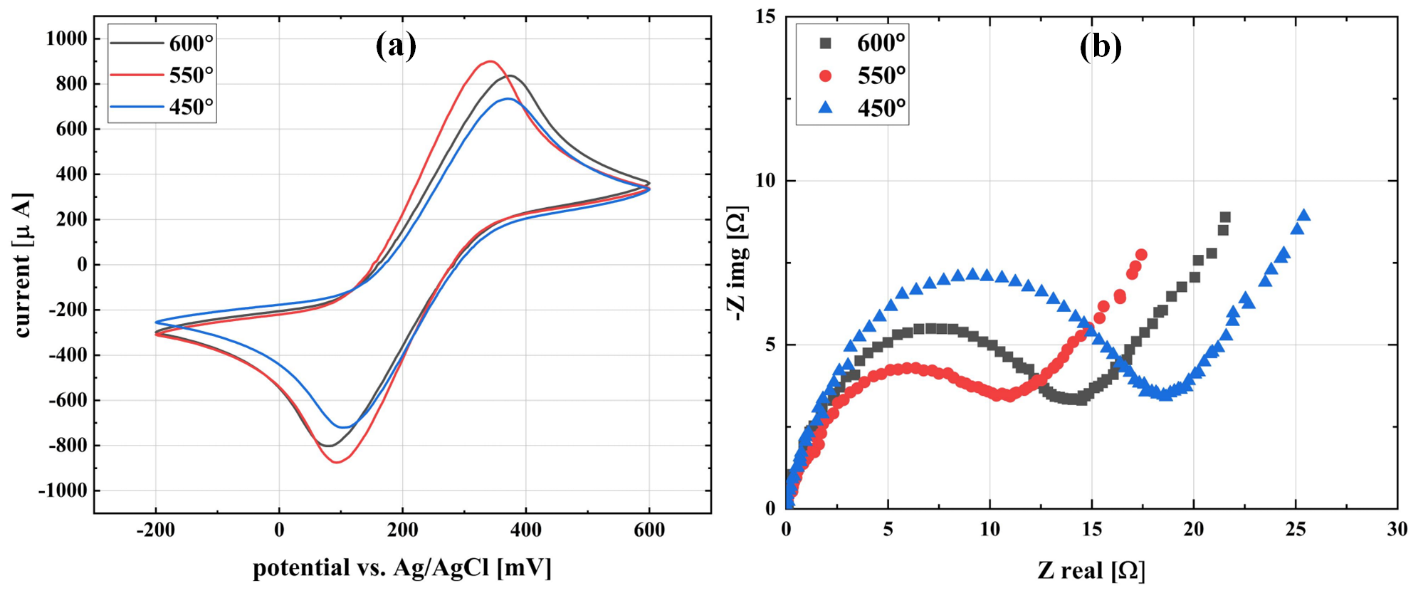


Figure.S3: The impact of the thermal annealing of the electrode on the CV (a), and EIS characterization results (b).


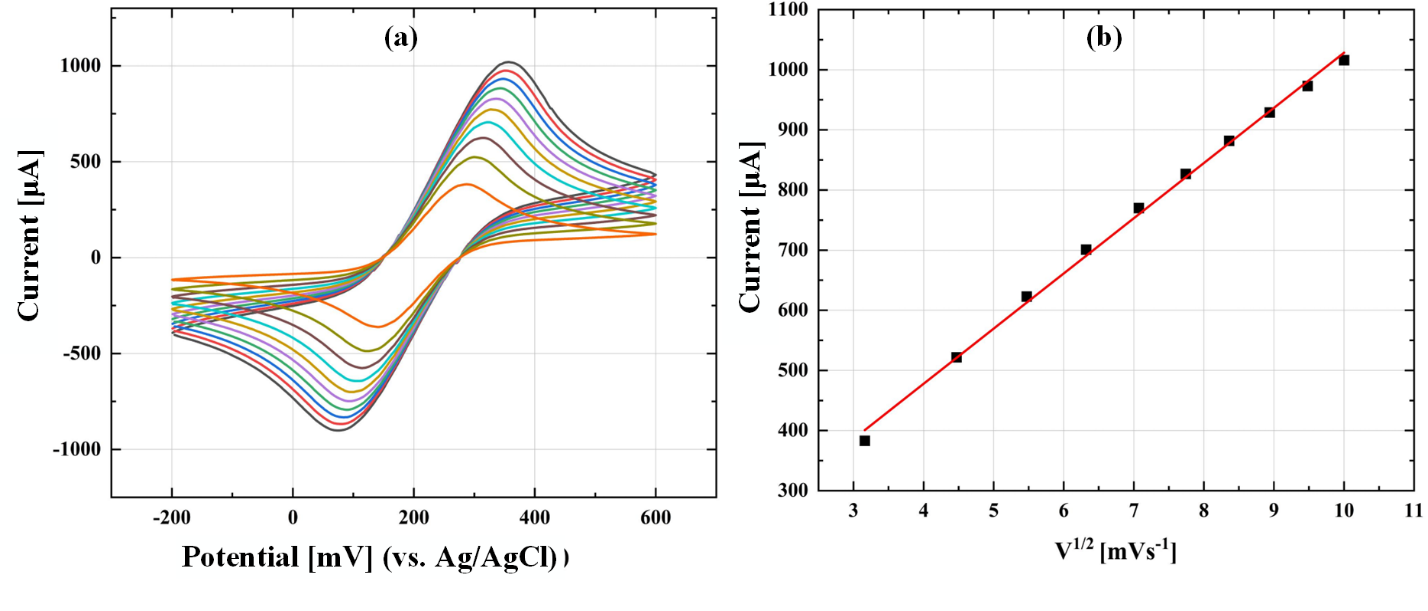


Figure.S4: CVs of the modified gold electrode at different scanning rates ranging from 10 to 100 mV/s.


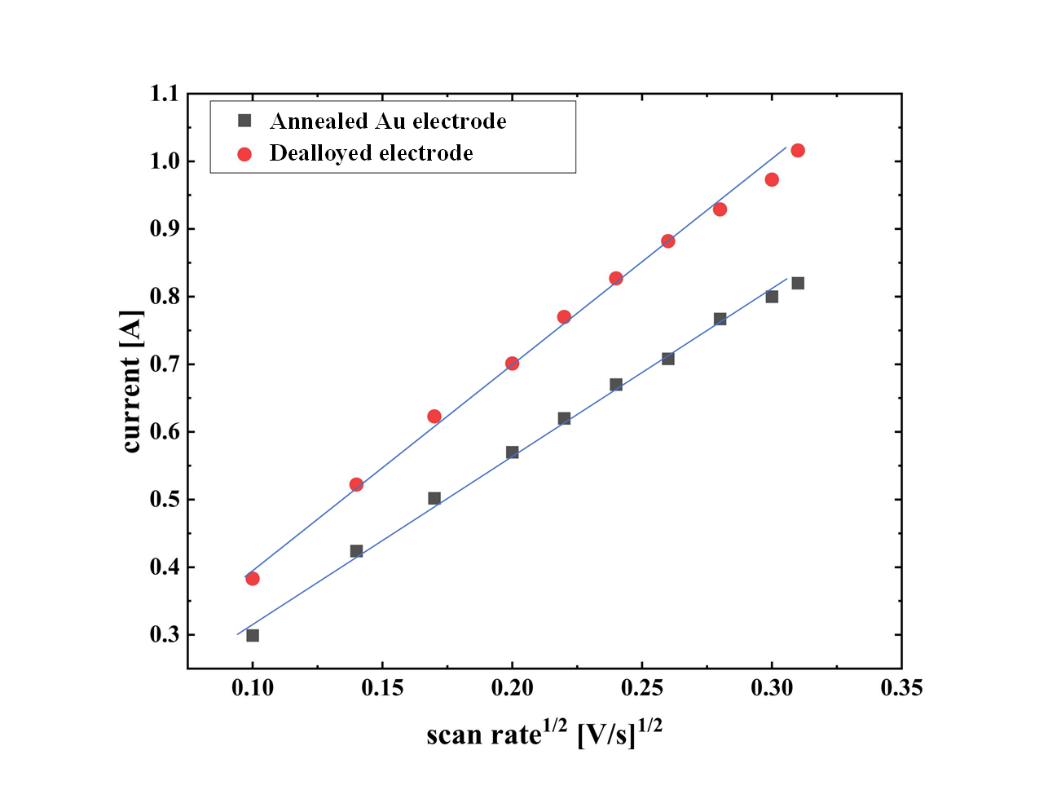


Figure.S5: The peak current as a function of the square root of the scan rate for both the annealed Au electrode and the dealloyed electrode, with scan rates ranging from 10 to 100 mV/s.

Table S1: A comparison of the effective surface areas between the annealed Au electrode and the dealloyed electrode.

| Electrode | Equation | R^2^ | S_effective_(Cm^2^) |
| --- | --- | --- | --- |
| Annealed Au electrode | I_pa_=2.44x+0.075 | 0.996 | 1.42 |
| Dealloyed electrode | I_pa_=2.94x+0.109 | 0.996 | 1.72 |


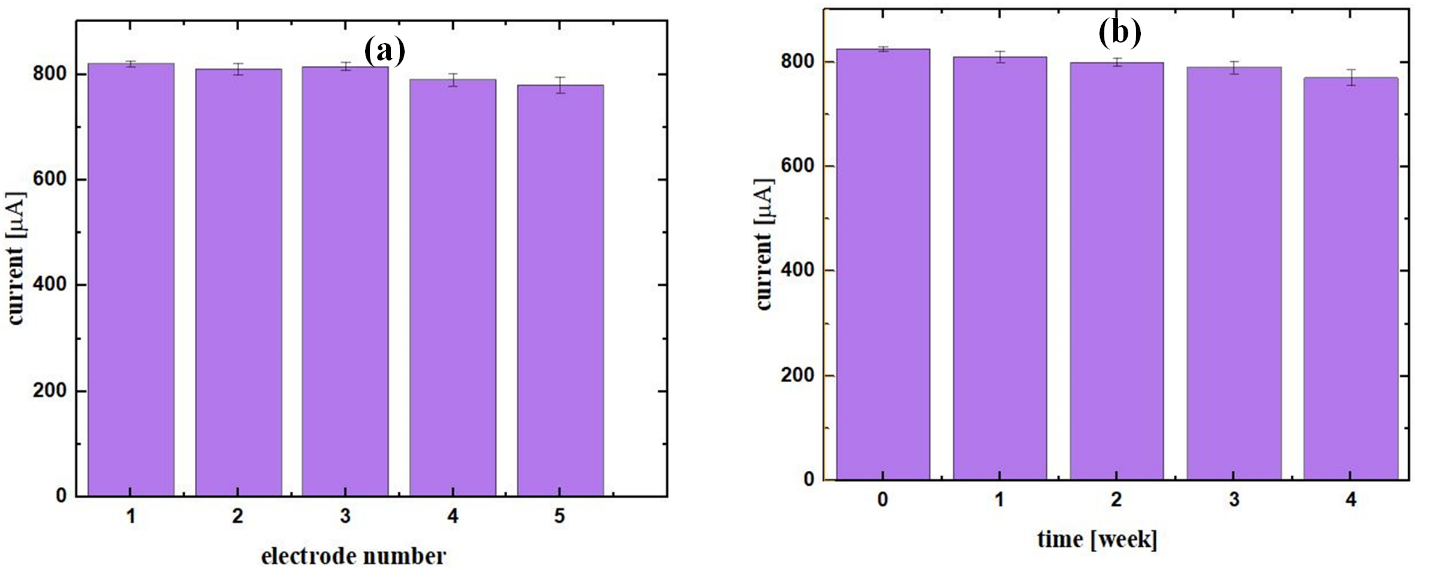
 Figure.S6: a) The CV peak current of 5 different electrode as reproducibility, and b) the time stability study of the immunosensor for four weeks

Table S2: Numerical values of the equivalent circuit components for the simulated immunosensor.

|  | R_S_ | R_ct_ | CPE (10^-5^ F) | $\alpha$ |
| --- | --- | --- | --- | --- |
| Bare | 66.7±4.1 | 15.8±2.2 | 8.8±1.14 | 0.82±0.01 |
| VHH | 79.2±3.3 | 59.5±4.1 | 6.5±0.51 | 0.83±0.01 |
| Block | 68.22±1.4 | 107±7.2 | 4.04±0.08 | 0.89±0.01 |
| VEGF  (10^-12^ gr/mL) | 89.2±1.7 | 205±8.1 | 1.75±0.05 | 0.92±0.01 |

Table S3: Determination of VEGF levels in both PBS and human blood serum samples as the results of recovery experiments for three known VEGF concentrations.

|  | In PBS | | | In serum | | |  |
| --- | --- | --- | --- | --- | --- | --- | --- |
| Samples | Actual concentration | Actual $\Delta R_{ct}$ | RSD  (n=3) | Observed concentration | Observed  $\Delta R_{ct}$ | RSD  (n=3) | Recovery |
| 1 | 1 pg/mL | 127.08 | 5% | 0.98 pg/mL | 126.4 | 4% | 98% |
| 2 | 100 pg/mL | 295.78 | 4% | 95.6 pg/mL | 294.1 | 3% | 95.6% |
| 3 | 1 ng/mL | 380.13 | 5% | 0.96 ng/mL | 378.6 | 6% | 96% |
